# Deletion of the GluA1 AMPA receptor subunit impairs recency-dependent object recognition memory

**DOI:** 10.1101/lm.2083411

**Published:** 2011-03

**Authors:** David J. Sanderson, Emma Hindley, Emily Smeaton, Nick Denny, Amy Taylor, Chris Barkus, Rolf Sprengel, Peter H. Seeburg, David M. Bannerman

**Affiliations:** 1 Department of Experimental Psychology, University of Oxford, South Parks Road, Oxford, OX1 3UD, United Kingdom; 2 Department of Molecular Neurobiology, Max-Planck Institute of Medical Research, D-69120 Heidelberg, Jahnstrasse 29, Germany

## Abstract

Deletion of the GluA1 AMPA receptor subunit impairs short-term spatial recognition memory. It has been suggested that short-term recognition depends upon memory caused by the recent presentation of a stimulus that is independent of contextual–retrieval processes. The aim of the present set of experiments was to test whether the role of GluA1 extends to nonspatial recognition memory. Wild-type and GluA1 knockout mice were tested on the standard object recognition task and a context-independent recognition task that required recency-dependent memory. In a first set of experiments it was found that GluA1 deletion failed to impair performance on either of the object recognition or recency-dependent tasks. However, GluA1 knockout mice displayed increased levels of exploration of the objects in both the sample and test phases compared to controls. In contrast, when the time that GluA1 knockout mice spent exploring the objects was yoked to control mice during the sample phase, it was found that GluA1 deletion now impaired performance on both the object recognition and the recency-dependent tasks. GluA1 deletion failed to impair performance on a context-dependent recognition task regardless of whether object exposure in knockout mice was yoked to controls or not. These results demonstrate that GluA1 is necessary for nonspatial as well as spatial recognition memory and plays an important role in recency-dependent memory processes.

The GluA1 subunit of the AMPA receptor is a key mediator of hippocampal synaptic plasticity (Zamanillo et al. 1999). It is especially important for a short-lasting, rapidly induced form of potentiation (Hoffman et al. 2002; Romberg et al. 2009; Erickson et al. 2010). We have recently shown that GluA1 knockout (GluA1^−/−^) mice display a deficit in hippocampus-dependent, short-term spatial recognition memory, but in contrast, show successful long-term spatial recognition memory (Sanderson et al. 2009). GluA1 deletion also impairs the expression of short-term visual recognition memory, but leaves long-term visual habituation intact (Sanderson et al. 2011). This suggests that short-term and long-term recognition memory are governed by separate processes (Wagner 1981; Sanderson et al. 2010; Sanderson and Bannerman 2011). A possible account is that short-term recognition may be caused by recency-dependent memory, and long-term recognition may be caused by an associative retrieval process whereby contextual cues aid recollection of the target stimulus (Wagner 1981).

In contrast to the impairment shown by GluA1^−/−^ mice on short-term, spatial recognition memory (Sanderson et al. 2007, 2009), a recent study has reported no effect of GluA1 deletion on object recognition (Wiedholz et al. 2008). This could reflect a difference between spatial and nonspatial stimulus processing. Alternatively, the lack of impairment could reflect the fact that there are two potential mechanisms that can support object recognition. For example, in a standard test of object recognition (e.g., Ennaceur and Delacour 1988), in which animals demonstrate greater exploration of a novel object over a preexposed, familiar object, memory may be caused by the recent presentation of the object during the exposure phase. Also, during the exposure phase the object and the context in which it is presented may become associated. In the test phase the context will be able to associatively retrieve a memory of the preexposed object such that recognition occurs. It is not possible to determine from the study of Wiedholz et al. (2008) whether object recognition was caused by recency-dependent memory or context-dependent memory. Therefore, it is possible that GluA1 may be necessary for object recognition but only when it relies on recency-dependent memory.

The aim of the present set of experiments was to assess (1) whether GluA1 is necessary for nonspatial object recognition as well as spatial recognition memory, and (2) whether GluA1 plays a role in, recency-dependent memory. Wild-type (WT) and GluA1^−/−^ mice were initially tested on the standard object recognition task in which they were exposed to two copies of an object (e.g., A) and then tested for their preference for a novel object (e.g., B) over the familiar object (i.e., A; see Fig. 1, top panel). Separate groups of mice were then tested on variants of the task that either minimized (object recency task) or maximized (context-dependent object recognition task) the use of context information.

**Figure 1.**
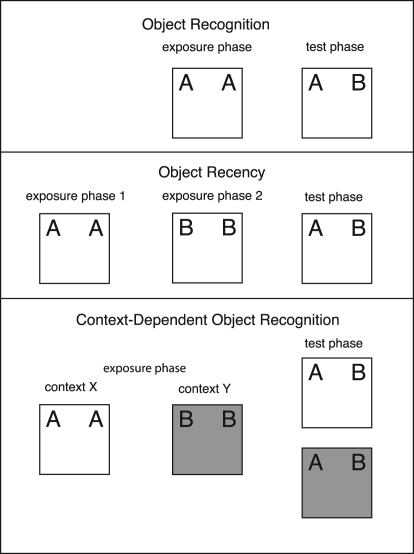
Design of the object recognition tasks. The *top* panel shows the design of the standard object recognition task. In the exposure phase (10 min) mice were exposed to two copies of an object and then after a 2-min interval mice received a 5-min test in which they were allowed to explore the familiar object and a novel object. The *middle* panel shows the design of the object recency task. In the exposure phase mice received two 10-min exposures to two different objects separated by a 2-min interval. The test phase (5 min) commenced 2 min after the last exposure. Mice were allowed to explore the more recently and the less recently presented objects. The *bottom* panel shows the design of the context-dependent object recognition task. In the exposure phase two different objects were exposed in two different contexts. Mice received four 10-min exposures to each object, one per day for 4 d. On the fifth day mice were simultaneously exposed to each object in each of the contexts in two 5-min tests.

In the object recency task (see Fig. 1, middle panel) mice are exposed to two copies of object A followed by two copies of object B, before receiving a test with A and B (e.g., Mitchell and Laiacona 1998; Hannesson et al. 2004; Good et al. 2007). In the test phase, the memory of the more recently presented object (i.e., B) may be stronger than the memory of the less recently presented object (i.e., A), because it has had less time to decay (e.g., Wagner 1981). Thus, mice should show a preference for object A over B. In the object recency task both objects are exposed and tested in the same context. Therefore, because each object has equal opportunity to form an association with the context, it is unlikely that context–object associations will aid performance in the test phase.

In the context-dependent object recognition task mice are exposed to the objects in two distinct contexts (e.g., Dix and Aggleton 1999; Mumby et al. 2002; Norman and Eacott 2005; Langston and Wood 2010). Object A is exposed in context X and object B is exposed in context Y (see Fig. 1, bottom panel). In the test trial mice are allowed to explore objects A and B in one of the contexts. The formation of an association between the context and the object in the exposure phase will result in mice showing a preference for exploring the object not previously paired with the context used in the test phase (i.e., context X: exploration of B > A; context Y: exploration of A > B). In the context-dependent task recognition can only be demonstrated by learning the associations between the specific contexts and the specific objects.

In Experiments 1 and 2 it was found that GluA1^−/−^ mice showed normal memory performance on the standard object recognition task and normal levels of recency-dependent recognition. However, GluA1^−/−^ mice demonstrated greater levels of object exploration during both exposure and test phases. In Experiments 3 and 4 the time that GluA1^−/−^ mice spent exploring the objects was yoked to that of WT mice, so that the two groups were matched. It was then found that GluA1^−/−^ mice were impaired on the object recognition and object recency tasks. GluA1 deletion failed to impair performance on the context-dependent recognition task, regardless of whether object exploration was yoked or not (Experiments 5 and 6). Thus, the results provide further evidence for the hypothesis that GluA1 is necessary for recency-dependent recognition memory.

## Results

### Experiment 1—Object recognition

In Experiment 1 female WT (*N* = 12) and GluA1^−/−^ (*N* = 12) mice were tested on the standard object recognition task. After habituation to the context (see Materials and Methods) mice were exposed to an identical pair of objects for 10 min and then, after a 2-min interval, they received a 5-min test in which they were allowed to explore the previously exposed, familiar object, and a novel object (see Fig. 1, top panel). Exploration was measured as time spent with the nose <1 cm away from an object (see Materials and Methods). Mice were removed from the analyses of the test phase if they failed to spend at least 1 sec exploring the objects during the test. A preference for the novel object over the familiar object indicates recognition memory.

#### Object exploration

**Figure 2.**
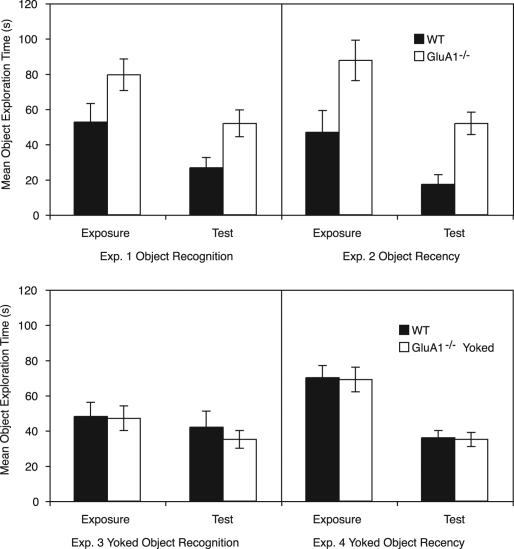
The mean object exploration times during the exposure and test phases of Experiments 1–4 for wild-type (WT) and GluA1^−/−^ mice. The *top left* panel shows the object exploration during the 10-min exposure phase and the 5-min test phase of the object recognition task (Experiment 1). The *top right* panel shows the object exploration for the object recency task (Experiment 2). The exposure object exploration is averaged across the two 10-min exposure trials. The *bottom left* panel shows the object exploration during the 10-min exposure phase and the 5-min test phase of the yoked object recognition task (Experiment 3). The object exploration for GluA1^−/−^ mice was yoked to that of WT mice. The *bottom right* panel shows the object exploration for the yoked object recency task (Experiment 4). The exposure object exploration is averaged across the two 10-min exposure trials. The object exploration for GluA1^−/−^ mice was yoked to that of WT mice. Error bars indicate ±SEM.

During the exposure phase there was a strong trend for GluA1^−/−^ mice to spend more time exploring the sample objects than WT mice (*F*_(1,22)_ = 3.80, *P* = 0.06, see Fig. 2, top left panel). In the test phase, this effect was found to be significant with GluA1^−/−^ mice showing greater combined exploration of the novel and familiar objects than WT mice (*F*_(1,22)_ = 6.88, *P* < 0.02, see Fig. 2, top left panel). One WT mouse spent <1 sec exploring the objects during the test phase, and therefore, was removed from the analyses of the test phase (WT: *N* = 11, see Materials and Methods).

#### Recognition test performance

In the test phase both groups spent more time exploring the novel object than the familiar object. The preference for the novel object was calculated as a discrimination ratio of the time spent exploring the novel object divided by the combined time spent exploring the novel and familiar objects during the test (i.e., novel/[novel + familiar]). Therefore, discrimination ratio scores >0.5 indicate a preference for the novel object.

**Figure 3.**
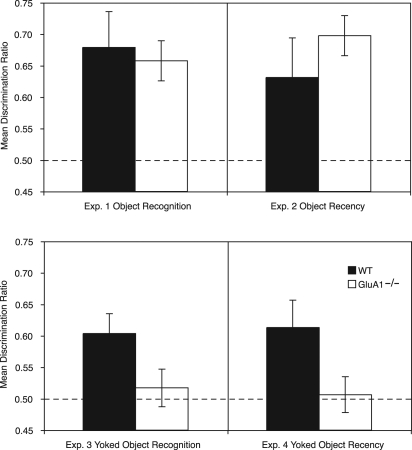
The results of the test phase for Experiments 1–4. The times spent exploring the novel object (Experiment 1, *top left* panel; Experiment 3, *bottom left* panel) and the less recently experienced object (Experiment 2, *top right* panel; Experiment 4, *bottom right* panel) are shown as a ratio of the total time spent exploring both objects in the respective test phases (see Results for details). The dashed line indicates chance performance. GluA1 deletion impaired performance on the object recognition (*bottom left* panel) and object recency (*bottom right* panel) tasks when the object exploration for GluA1^−/−^ mice was yoked to that of WT mice. Error bars indicate ±SEM.

Analysis of the discrimination ratios demonstrated that both groups showed a similar preference for the novel object (see Fig. 3, top left panel) that did not significantly differ from each other (*F* < 1). Further analysis confirmed that the preference for the novel object was significantly above chance for both groups (WT: *t*_(10)_ = 3.13, *P* < 0.01; GluA1^−/−^: *t*_(11)_ = 3.52, *P* < 0.003, see Fig. 3, top left panel).

### Experiment 2—Object recency

In Experiment 2 WT (female, *N* = 6; male, *N* = 6) and GluA1^−/−^ (female, *N* = 6; male, *N* = 6) mice were tested on the object recency task (see Fig. 1, middle panel). After habituation to the context (see Materials and Methods) mice were initially exposed to two copies of object A for 10 min and then after a 2-min interval they received a 10-min exposure to two copies of object B. After a further 2-min interval mice received a test in which they were allowed to explore both objects A and B (see Fig. 1, middle panel). A preference for object A over object B indicates greater memory for the more recently experienced object than for the less recently experienced object. Whereas performance on the standard object recognition task (Experiment 1) may reflect both recency-dependent and context-dependent memory processes, performance on the object recency task can only reflect recency-dependent memory.

#### Object exploration

During the two exposure phases it was found that the GluA1^−/−^ mice again explored the objects significantly more than the WT mice (*F*_(1,20)_ = 5.93, *P* < 0.03, see Fig. 2, top right panel). It was similarly found that GluA1^−/−^ mice showed greater object exploration than WT mice in the test phase (*F*_(1,20)_ = 17.01, *P* < 0.002, see Fig. 2, top right panel). There was no significant effect of sex, and no significant interaction between sex and genotype for analyses of both the exposure phase and the test phase (all *P* values >0.2). Also, in the analysis of the exposure phase, the effect of exposure trial (i.e., exposure phase 1 vs. exposure phase 2) was not significant and did not significantly interact with other factors (all *P* values >0.2). In the test phase three male WT mice spent <1 sec exploring the objects and were thus removed from the analysis of the discrimination ratios (WT: *N* = 9).

#### Recognition test performance

In the test phase both groups showed greater exploration of the less recently presented object than for the more recently presented object. The preference for the less recently presented object was calculated as a discrimination ratio of the time spent exploring the less recently presented object (i.e., first object) divided by the total time spent exploring the two objects (i.e., first object/[first object + second object]).

Analysis of the discriminations ratios demonstrated that WT and GluA1^−/−^ mice did not significantly differ in their preference for the less recently exposed object (*F* < 1, see Fig. 3, top right panel). Moreover, both the WT and GluA1^−/−^ mice showed a significant preference for the less recently exposed object (WT, *t*_(8)_ = 2.09, *P* < 0.04; GluA1^−/−^, *t*_(11)_ = 2.7, *P* = 0.01). There was no significant effect of sex or interaction of factors (all *P* values >0.2).

### Experiment 3—Yoked object recognition

In Experiments 1 and 2 GluA1 deletion failed to impair recognition memory performance. However, it was found that GluA1 deletion increased levels of object exploration. It is possible that the increased object exploration during the exposure phase may have resulted in greater opportunity for learning that may have compensated for any deficit in GluA1^−/−^ mice.^4^ Experiments 3 and 4 removed this confound by yoking the exploration of GluA1^−/−^ mice to that of WT mice (see Materials and Methods) for all phases of the experiments.

In Experiment 3 WT (female, *N* = 6; male, *N* = 6) and GluA1^−/−^ (female, *N* = 6; male, *N* = 6) mice were tested on the object recognition task in a similar manner to Experiment 1 (see Fig. 1, top panel). However, now the time that GluA1^−/−^ mice spent exploring the objects in both the exposure and test phases were yoked to WT mice. Yoking was achieved by running pairs of WT and GluA1^−/−^ mice and terminating a trial for a GluA1^−/−^ mouse once it had accumulated the same amount of object exploration as the equivalently run WT mouse, or had otherwise reached the time limits of the exposure and test phases (see Materials and Methods).

#### Object exploration

One female WT mouse spent <1 sec exploring the objects during the test phase. Therefore, the data for this mouse and its paired GluA1^−/−^ mouse were removed from the subsequent analyses (i.e., leaving *N* = 11 per genotype).

The levels of object exploration for WT mice in the exposure and test phases are shown in Figure 2, bottom left panel. There was no significant effect of sex in either stage (*P* values >0.1).

During the exposure and test phases GluA1^−/−^ mice took less time to accumulate the same duration of total object exploration as WT mice. This was tested using one-sample *t*-tests to compare the duration of the exposure and test phases of the GluA1^−/−^ mice to the fixed duration of the exposure and test phases for WT mice (i.e., 10 min and 5 min, respectively; exposure phase, GluA1^−/−^ mean = 3.98 min ± 0.7 SEM, *t*_(10)_ = 8.61, *P* < 0.0005; test phase, GluA1^−/−^ mean = 3.13 min ± 0.49 SEM, *t*_(10)_ = 3.79, *P* < 0.0025). Although one GluA1^−/−^ mouse in the exposure phase and two GluA1^−/−^ mice in the test phase did not accumulate the required exposure times of their paired WT mice, overall, object exploration did not significantly differ between the groups (exposure and test phase, *F* values <1, see Fig. 2, bottom right panel).

#### Recognition test performance

Importantly, during the test phase WT mice now showed a significantly greater preference for the novel object than GluA1^−/−^ mice (*F*_(1,18)_ = 4.63, *P* < 0.05, see Fig. 3, bottom left panel). The WT mice showed a novelty preference that was significantly greater than chance (*t*_(10)_ = 3.28, *P* < 0.005, but the GluA1^−/−^ mice did not (*t* < 1). Neither the effect of sex nor the interaction were significant (*P* values >0.15).

### Experiment 4—Yoked object recency

In Experiment 4 WT (female, *N* = 12; male, *N* = 12) and GluA1^−/−^ (female, *N* = 12; male, *N* = 12) mice were tested on the object recency task (see Fig. 1, middle panel) in a similar manner to Experiment 2. However, now the times that GluA1^−/−^ mice spent exploring the objects in the exposure and test phases were yoked to that of WT mice (see Experiment 3 Results and Methods for further details).

#### Object exploration

Three WT mice (two female, one male) spent <1 sec exploring either of the objects during the test. These mice and the corresponding yoked GluA1^−/−^ mice were removed from the analyses of both exposure and test phases.

The levels of object exploration for WT mice in the exposure and test phases are shown in Figure 2, bottom right panel. For the exposure phase there was no significant effects of exposure trial, sex, or interactions of factors (all *P* values >0.2). There was no significant effect of sex (*F* < 1) in the test phase.

Two GluA1^−/−^ mice in the first exposure phase and one GluA1^−/−^ mouse in the test phase failed to accumulate the required exposure times of their paired WT mice. However, overall, object exploration did not significantly differ between the groups (exposure phases and test phase, *F* values <1, see Fig. 2, bottom right panel).

Again, as in Experiment 3, during both the exposure and test phases the GluA1^−/−^ mice took less time to accumulate the same duration of object exploration, in the exposure and test phases, as WT mice (i.e., 10 min and 5 min, respectively). One-sample *t*-tests confirmed that this effect was significant (first exposure, GluA1^−/−^ mean = 5.17 min ± 0.56 SEM, *t*_(20)_ = 8.62, *P* < 0.0005; second exposure, GluA1^−/−^ mean = 5.74 min ± 0.54 SEM, *t*_(20)_ = 7.88, *P* < 0.0005; test phase, GluA1^−/−^ mean = 2.76 min ± 0.28 SEM, *t*_(20)_ = 8.06, *P* < 0.0005).

#### Recognition test performance

**Figure 4.**
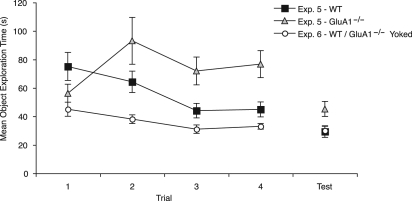
The mean object exploration across the exposure phase in Experiments 5 and 6. The black squares and the gray triangles show the exploration of the WT and GluA1^−/−^ mice, respectively, in Experiment 5. The white circles show the exploration of the WT mice in Experiment 6. The object exploration for GluA1^−/−^ mice, in Experiment 6, was yoked to that of WT mice. Data for the four exposure trials are shown averaged across the two different objects. Error bars indicate ±SEM.

During the test phase WT mice showed a significantly greater preference for the less recent object than GluA1^−/−^ mice (*F*_(1,38)_ = 4.35, *P* < 0.05, see Fig. 3, bottom right panel). Furthermore, the WT mice showed a preference for the less recent object that was significantly greater than chance (*t*_(20)_ = 2.62, *P* < 0.01), but the GluA1^−/−^ mice did not (*t* < 1, see Fig. 4). Neither the effect of sex or interaction were significant (*F* values <1).

### Experiment 5—Context-dependent object recognition

In Experiment 4 GluA1 deletion impaired memory when performance in the WT mice could only be due to recency-dependent processes. However, in Experiment 3, GluA1 deletion also impaired memory when performance could be due to either recency-dependent or context-dependent processes, or indeed, the sum of these two processes. It is possible that performance in WT mice in the standard object recognition task (Experiment 3) relied on recency-dependent memory, and thus GluA1 deletion had a similar effect in both Experiments 3 and 4. One reason for suspecting that performance in the standard object recognition task relied on recency-dependent memory is that the levels of performance in WT mice in the object recognition task (Experiments 1 and 3) and the object recency task (Experiments 2 and 4; see Fig. 3) did not significantly differ (*F* < 1). This suggests that there was no additive effect of context-dependent information in the object recognition task.

Context-dependent processes may have failed to aid recognition in the object recognition task for two possible reasons. First, there may have been insufficient exposure to the context for context–object associations to have formed. Second, the preexposure to the context prior to exposure to the objects may have resulted in latent inhibition, thus retarding context–object learning.

The purpose of Experiment 5 was to assess whether GluA1 is necessary for context-dependent recognition by training mice on a task that maximized the use of contextual information, and required learning context–object associations for performance in the test phase. To ensure that WT and GluA1^−/−^ mice had equal exposure to the contextual information, object exploration was not yoked, and thus trial lengths were equal for both groups.

WT (female, *N* = 4; male, *N* = 4) and GluA1^−/−^ (female, *N* = 4; male, *N* = 4) mice were tested on the context-dependent object recognition task (see Fig. 1, bottom panel). Mice were exposed to objects in two distinct contexts. Object A was exposed in context X and object B was exposed in context Y (see Materials and Methods). Mice received four 10-min exposures to each context, one exposure per context, per day. On the fifth day mice received two 5-min test trials in which they were exposed to both objects A and B simultaneously, in each context (i.e., X and Y, see Fig. 1, bottom panel). In these test trials, a preference for the object that was not previously paired with the context (unpaired object) indicates context-dependent object recognition memory.

In contrast to the previous experiments mice received four times as much exposure to the objects so as to increase the likelihood of the formation of context–object associations. The exposure occurred across repeated trials so as to aid incremental strengthening of memory (Sanderson and Bannerman 2011). Also, mice were not preexposed to the contexts (prior to object exploration) so as to eliminate the possibility of latent inhibition occurring. To reduce the influence of recency-dependent memory in the test phase, mice received the recognition test 24 h after the last exposure trial. To confirm whether or not this was case the relative recency of the unpaired object on the first test trial (based on the order of exposure trials on the day prior to the test phase) was included as a factor in the analysis of the discrimination ratios (see Materials and Methods for details of the counterbalancing). Thus, if the unpaired object in the first test trial was presented less recently than the paired object it might be predicted that the preference for the unpaired object would be higher than if the unpaired object was presented more recently than the paired object.

#### Object exploration

The object exploration during the exposure training phase was analyzed using a 2 (Genotype: WT, GluA1^−/−^) × 2 (Sex: male, female) × 2 (Context: X, Y) × 4 (Exposure Trial: 1–4) ANOVA. Although the main effect of genotype failed to reach significance (*F*_(1,12)_ = 3.54, *P* = 0.08) there was a significant Genotype× Trial interaction (*F*_(3,36)_ = 7.07, *P* < 0.002, see Fig. 4). Notably, simple main effects analysis demonstrated that GluA1^−/−^ mice showed less exploration of objects than WT mice on exposure trial 1 (*F*_(1,12)_ = 6.21, *P* < 0.03), but they showed greater object exploration than WT mice on exposure trials 3 and 4 (*P* values <0.02). The two genotypes did not significantly differ on trial 2 (*P* > 0.1). It was also found that, overall, mice spent more time exploring objects in context X than in context Y (*F*_(1,12)_ = 22.14, *P* < 0.002) and female mice showed greater exploration than male mice (*F*_(1,12)_ = 5.68, *P* < 0.05). There were no other significant interactions between factors (all *P* values >0.08).

During the test trials it was once again found that, overall, GluA1^−/−^ mice explored the objects more than WT mice (*F*_(1,12)_ = 5.04, *P* < 0.05, see Fig. 4). There was no significant effect of sex (*F* < 1) or test trial (*F* < 1) or any interactions between factors (all *P* values >0.27).

#### Recognition test performance

In the test phase both groups showed greater exploration of the unpaired object than the paired object. Preference for the unpaired object was calculated as a discrimination ratio of the time spent exploring the unpaired object divided by the combined time spent exploring the two objects (i.e., unpaired/[unpaired + paired]). Data from the two test trials (i.e., from both contexts) were combined to calculate the discrimination ratios.

**Figure 5.**
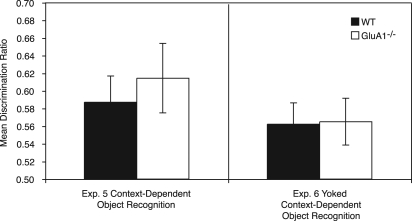
The results of the test phase for Experiments 5 and 6. Time spent exploring the object not previously paired with the test context (i.e., the unpaired object) is shown as a ratio of the total time spent exploring the paired and unpaired objects. A score of 0.5 indicates chance performance. In Experiment 5 (*left* panel) WT and GluA1^−/−^ mice were allowed to freely explore objects for the duration of each trial. In Experiment 6 (*right* panel) the object exploration for GluA1^−/−^ mice was yoked to that of WT mice. Error bars indicate ±SEM.

WT and GluA1^−/−^ mice showed a similar preference for the unpaired object and did not significantly differ from one another (*F* < 1, see Fig. 5, left panel). The preference was significantly above chance for both groups (WT, *t*_(7)_ = 2.91, *P* < 0.02; GluA1^−/−^, *t*_(7)_ = 2.91, *P* < 0.02). The relative recency of the unpaired object failed to affect performance (*F* < 1). Thus, there was no evidence of recency-dependent memory after a 24-h interval. There was no significant effect of sex (*P* > 0.1) and no significant interaction of factors (*P* values ≥0.3).

### Experiment 6—Yoked context-dependent object recognition

In Experiment 5 GluA1 deletion did not impair context-dependent object recognition. However, the levels of object exploration differed between the WT and GluA1^−/−^ mice. In Experiment 6, to rule out the possibility that greater object exploration compensated for a potential deficit in GluA1^−/−^ mice, naïve WT (female, *N* = 8; male, *N* = 8) and GluA1^−/−^ (female, *N* = 8; male, *N* = 8) mice were tested on the context-dependent object recognition task (see Fig. 1, bottom panel), but now the time that GluA1^−/−^ mice spent exploring the objects in all of the exposure and test phases were yoked to that of WT mice (see Materials and Methods for further details). In contrast to Experiments 3 and 4, there was now no cutoff time for GluA1^−/−^ mice in exposure and test trials. Therefore, trials for GluA1^−/−^ mice were only terminated once they had accumulated the same amount of object exploration as their paired WT mouse.

#### Object exploration

The levels of object exploration over trials for WT mice (and consequently for the yoked exploration for GluA1^−/−^ mice) are shown in Figure 4. Similar to Experiment 5, WT mice showed a decline in object exploration over repeated trials *F*_(3,42)_ = 7.71, *P* < 0.0005). This effect significantly interacted with sex (*F*_(3,42)_ =4.7, *P* < 0.007) due to male mice initially showing greater levels of object exploration than female mice. The effect of context was not significant (*F*_(1,14)_ = 3.26, *P* > 0.09) and there were no other significant interactions between factors (*P* values >0.1).

The levels of object exploration for WT mice (and consequently for the yoked exploration for GluA1^−/−^ mice) in the test phase are shown in Figure 4. The factors of sex and trial, and the interactions were not significant (all *F* values <1).

Across exposure trials GluA1^−/−^ mice took, on average, less time to accumulate the same level of object exploration as WT mice (i.e., WT = 10 min, GluA1^−/−^ mean = 7.84 min ± 0.67 SEM, *t*_(15)_ = 3.23, *P* < 0.007). This was also true for the test trials (i.e., WT = 5 min; test trial 1, GluA1^−/−^ mean = 3.66 min ± 0.57 SEM, *t*_(15)_ = 2.37, *P* < 0.04; test trial 2, GluA1^−/−^ mean = 3.69 min ± 0.48 SEM, *t*_(15)_ = 2.71, *P* < 0.02).

#### Recognition test performance

Similar to the results of Experiment 5, both groups showed greater exploration of the unpaired object than the paired object. To analyze the preferences for the unpaired object, the data from the two test trials were combined for the analysis of the discrimination ratios. For GluA1^−/−^ mice discrimination ratios were calculated from the combined yoked periods of object exploration during each test trial.

WT and GluA1^−/−^ mice both showed a similar preference for the unpaired object that did not significantly differ (*F* < 1, see Fig. 5, right panel). The preference for the unpaired object was significantly above chance for both groups (WT, *t*_(15)_ = 2.56, *P* < 0.02; GluA1^−/−^ (*t*_(15)_ = 2.45, *P* < 0.02). As in Experiment 5, there was no significant effect of relative recency (*F* < 1), thus failing to demonstrate recency-dependent memory after a 24-h interval. There was no significant effect of sex (*P* > 0.1) or significant interactions (*F* values <1).

## Discussion

The results of Experiments 1 and 2 suggested that GluA1^−/−^ mice were not impaired on the object recognition or object recency tasks. However, GluA1^−/−^ mice also showed greater levels of object exploration than WT mice, during both exposure and test phases. Increased object exploration during the exposure phase could have compensated for any learning deficit in these animals, resulting in a rescue of memory performance. Therefore, in Experiments 3 and 4 the confound of different levels of exploration was removed by yoking the amount of object exploration in GluA1^−/−^ mice to that of WT mice. It was now found that GluA1^−/−^ mice were impaired, both on the standard object recognition task and the object recency task.

These results demonstrate that the GluA1 subunit of the AMPA glutamate receptor plays an important role in nonspatial, as well as spatial, recognition memory (Sanderson et al. 2007, 2009; see also Sanderson et al. 2011). A previous investigation of object recognition in GluA1^−/−^ mice had failed to find a deficit (Wiedholz et al. 2008). In this study mice received repeated exposures to an array of objects before receiving a test trial in which one of the preexposed, familiar objects was replaced with a novel object. Mice were allowed to freely explore the objects during the exposure trials and it was found that, consistent with the present study, GluA1^−/−^ mice explored the objects more than WT mice. Both WT and GluA1^−/−^ mice then demonstrated a robust and equivalent preference for the novel object (Wiedholz et al. 2008). Consistent with this previous report, there was no deficit in the GluA1^−/−^ mice in Experiment 1 of the present study in which the exploration of GluA1^−/−^ mice was not yoked to that of WT mice. Thus, GluA1^−/−^ mice showed apparently normal object recognition memory. However, they also displayed increased object exploration during both the exposure and test phases. The fact that GluA1^−/−^ mice showed greater levels of object exploration than WT mice, both in Experiment 1 and in the study by Weidholz et al. (2008), could be indicative of a failure of the GluA1^−/−^ mice to show recency-dependent recognition of the objects. Furthermore, this increase in object exploration might allow greater opportunity for learning and thus could potentially compensate for any memory deficit in these animals. Indeed, in view of the subsequent results of Experiment 3, in which object exploration was yoked, it seems likely that this initial failure to find an effect of GluA1 deletion on object recognition was due to the increased levels of exploration during the exposure phase, masking a deficit in GluA1^−/−^ mice.

### Recency-dependent and context-dependent recognition memory

The different recognition memory tasks (i.e., object recognition, object recency, and context-dependent object recognition) provide a potential means of examining the psychological processes underlying recognition. Although performance on the object recognition task (Experiment 3) could plausibly reflect the use of either recency-dependent or context-dependent information, performance on the object recency task (Experiment 4) reflects the use of recency-dependent information. The fact that WT mice showed similar levels of performance in the object recognition task (Experiments 1 and 3) and the object recency task (Experiments 2 and 4) suggests that, in fact, the context did not aid retrieval in the test phase for the object recognition task. Thus, the impairments caused by GluA1 deletion in both Experiments 3 and 4 are likely to reflect impaired recency-dependent memory.

In Experiments 5 and 6 WT mice demonstrated context-dependent recognition when given sufficient exposure training. Importantly GluA1^−/−^ mice were not impaired regardless of whether exploration was yoked or not. Thus, even when GluA1^−/−^ mice received less exposure to the contexts than WT mice (Experiment 6) performance was not impaired. These results demonstrate the GluA1^−/−^ mice can show normal discrimination between the objects when object exploration is matched to controls. Also, the impairments in Experiments 3 and 4 are unlikely to be due to a nonspecific effect of the yoking procedure, because performance was normal in the yoked context-dependent object recognition task (Experiment 6).

### A possible role for GluA1 in recency-dependent memory

Taken together the impaired performance in Experiment 4 and the normal performance in Experiment 6 suggests that GluA1 is necessary for recency-dependent, but not context-dependent recognition. However, in addition to the psychological demands of the respective tasks, the experiments also differed in the amount of exposure to the objects. Thus, it is possible that GluA1 deletion impairs object recognition depending on the level of exposure to stimuli, rather than on the psychological processes required for memory retrieval. There are several reasons for favoring an account based on GluA1 deletion selectively impairing recency-dependent memory.

First, GluA1 deletion impairs the expression of recency-dependent visual recognition memory in a task in which mice received extended exposure to the stimuli (Sanderson et al. 2011). In this task recency-dependent memory was repeatedly assessed over time, using two distinct lights presented in an operant box that became increasingly familiar over training. The effect of GluA1 deletion was consistent throughout testing demonstrating that it was not dependent on the level of stimulus exposure. Furthermore, this recency-dependent impairment in GluA1^−/−^ mice remained constant even though both groups showed a similar long-term reduction in responding to the stimuli over training.

Second, GluA1 deletion impairs short-term, recency-dependent spatial recognition memory, but actually enhances long-term spatial recognition memory (Sanderson et al. 2009). Specifically, when exposure trials were separated by a short interval (massed exposure) GluA1^−/−^ mice were impaired, but when exposures were separated by a long interval (spaced exposure) GluA1^−/−^ mice were facilitated. These effects occurred even though the massed exposure training elicited greater levels of activity than the spaced exposure in GluA1^−/−^ mice. Thus, the pattern of results in this study cannot be readily explained by the level of exposure to the spatial stimuli. The opposite effects of GluA1 deletion on spatial recognition when exposure is either massed or spaced likely reflects competition between nonassociative, recency-dependent memory and associative, context-dependent memory (see Sanderson and Bannerman 2011).

Therefore, the effects of GluA1 deletion on visual and spatial recognition demonstrate that GluA1 plays a role in recognition memory that is independent of not only the amount of stimulus exposure that mice receive, but also the modality of the stimuli. Therefore, it may be parsimonious to consider psychological accounts of the present results with object stimuli that accommodate the previous findings with stimuli from different modalities (Sanderson et al. 2009, 2011).

### A dual-process memory model

A model of memory proposed by Wagner (1981) provides a potential account of the role of GluA1 in recognition memory. Wagner proposed that a stimulus is represented by a set of elements. Elements can reside in one of three memory states. When a stimulus is presented it increasingly activates its elements into a primary activity state (A1). From the A1 state elements rapidly decay into a secondary activity state (A2) before gradually decaying into the inactive state. Whereas elements in the A1 state can elicit a high level of responding, elements in the A2 state are less able to elicit responding. Elements in the A2 state also cannot reenter the A1 state if the stimulus is subsequently presented. A consequence of this is that if the elements of a stimulus representation are in the A2 state when the stimulus is subsequently presented then the stimulus will be less able to elicit responding. However, if enough time has passed such that the representation has returned to the inactive state, then the stimulus will be able to fully activate its elements into the A1 state. Thus, a more recently presented stimulus will elicit less responding than a less recently presented stimulus. This description of memory provides an account of the behavior of WT mice in the object recency task, in which they showed greater exploration of the less recently experienced object than the more recently explored object.

Wagner also suggested that elements can be directly activated to the A2 state from the inactive state by a process of associative retrieval. Thus, if two stimuli, X and Y, have formed an association such that X predicts the occurrence of Y, then presentation of X will lead to activation of elements of Y into the A2 state. Thus, an associatively retrieved stimulus will elicit less unconditioned responding than a stimulus that has not been associatively retrieved. The proportion of elements that are activated into the A2 state depends on the strength of association between stimuli. Therefore, in contrast to the recency-dependent A2 activation, associative retrieval can have a long-term influence on responding. This description of memory provides an account of the behavior of mice in context-dependent object recognition experiments. Thus, mice explored the unpaired object (that was previously not associated with the test context) more than the paired object.

By applying Wagner's model to the present results with objects, it would suggest that GluA1 is necessary for A2 activation that occurs as a result of a recent stimulus presentation, but not as a result of associative retrieval. Thus, GluA1 deletion impairs recency-dependent recognition (Experiment 4), but not associative, context-dependent object recognition (Experiment 6). According to this analysis GluA1 deletion should impair short-term memory, but not long-term memory. The effect of GluA1 deletion on visual memory using punctate lights in an operant box are consistent with this hypothesis. GluA1 deletion impaired short-term, recency-dependent visual memory despite leaving long-term habituation of the visual cues intact (Sanderson et al. 2011). Also, GluA1 deletion impairs the ability to discriminate between spatial locations on the basis of their relative recency in spatial win-shift tasks, but does not impair long-term spatial reference memory performance (Reisel et al. 2002; Schmitt et al. 2003).

Wagner's model also makes the prediction that short-term memory can compete with long-term memory under certain conditions. Associations that underlie long-term memory are formed between elements of stimuli that are concurrently active in the A1 state. If the interval between presentations of a stimulus is short such that the stimulus’ elements are in the A2 state when it is subsequently presented, then there will be fewer elements that are able to enter the A1 state. Consequently, there will be a reduction in the ability of the stimulus to enter associations with other cues in the environment (e.g., Best and Gemberling 1977; Sunsay et al. 2004). A prediction that follows from Wagner's model is that a reduction in short-term, recency-dependent memory may allow for greater long-term learning. The effects of GluA1 deletion on spatial recognition memory are consistent with this claim. GluA1 deletion enhanced long-term spatial recognition memory, despite impairing short-term spatial recognition memory (Sanderson et al. 2009). Collectively, the findings with visual and spatial stimuli, and the present results with objects, provide converging evidence that GluA1 is important for recency-dependent recognition.

### The role of synaptic plasticity in recency-dependent memory

Given the specific role of GluA1 in short-term recognition memory, it is tempting to speculate on a role for a rapidly induced, short-lasting, GluA1-dependent form of synaptic plasticity in this process. However, while most electrophysiological studies examining the role of GluA1 in synaptic plasticity have concentrated on its role in potentiation of pyramidal cell activity (Zamanillo et al. 1999; Hoffman et al. 2002; Romberg et al. 2009), this may not be the most plausible substrate for recency-dependent, short-term recognition memory. It seems more likely that when a stimulus representation enters the A2 state, it is due to a reduction in the excitability of the appropriate neuronal ensemble. Consistent with this hypothesis there is considerable evidence from human fMRI studies that neuronal activity is reduced when stimuli are repeated, compared to when a novel stimulus is presented (e.g., Henson et al. 2003; Ranganath and Rainer 2003; Grill-Spector et al. 2006; Kumaran and Maguire 2006). Furthermore, studies in monkeys have shown that neurons in the perirhinal cortex, a brain region that is strongly implicated in object recognition, suppress their firing to repetitions of stimuli (Brown et al. 1987; Brown and Aggleton 2001). Therefore, GluA1 may play an important role in the short-term, recency-dependent reduction of neuronal excitability following a stimulus presentation, although the mechanism by which this might occur is not known. Nevertheless, regardless of the mechanism, it is clear that there are separate GluA1-dependent and GluA1-independent processes that contribute to the neural basis of recognition memory.

### Conclusions

We have previously shown that GluA1 deletion impairs short-term, recency-dependent spatial and visual recognition memory (Sanderson et al. 2009, 2011). We now demonstrate that GluA1 deletion similarly impairs recency-dependent memory for nonspatial, object stimuli. This is in contrast to the normal performance of GluA1^−/−^ mice on a context-dependent object recognition task. Collectively, the results of Sanderson et al. (2009, 2011), and the present experiments are potentially accommodated by a model of learning that proposes that short-term and long-term memory reflect nonassociative and associative processes respectively (Wagner 1981).

## Materials and Methods

### Subjects

Experimentally naïve, littermate, age-matched, male and female, WT and GluA1^−/−^ mice, bred in the Department of Experimental Psychology, University of Oxford, served as subjects in these experiments (for details of genetic construction, breeding, and subsequent genotyping, see Zamanillo et al. 1999). Mice were caged in groups of two to six, in a temperature controlled housing room on a 12-h light/dark cycle (0700–1900), and had ad libitum access to food and water. Mice were between 6 and 10 months at the time of testing. All procedures were in accordance with the United Kingdom Animals Scientific Procedures Act (1986); under project license number PPL 30/2561.

### Apparatus

A total of four objects were used for the experiments: tin can, plastic bottle, glass bottle, and wooden block. Pilot work had demonstrated that the four objects elicited equal levels of exploration. There were four copies of each object. All objects were sufficiently heavy so that they could not to be displaced by the mice. In Experiment 1 all four objects were used, but Experiments 2–6 used only the plastic bottle and the wooden block.

In Experiments 1, 2, 3, and 4 the test arena was a square, gray wooden box (40 × 40 × 40 cm). In Experiments 5 and 6 two test arenas were used so that mice could be tested in two separate contexts. Context X was the gray wooden box used in Experiments 1, 2, 3, and 4, and context Y was a white plastic box (50 × 50 × 50 cm) that had a black grid painted on the floor of the box. A laminated black and white striped card was attached to the walls of the box. The two boxes were located in separate testing rooms that contained distinct spatial and odor cues. In each test room a camera was suspended from the ceiling directly above the center of the arena. For context X the camera was connected to a computer, located in an adjacent room, which used Ethovision XT (Noldus) to record the trials. For context Y the camera was connected to a DVD recorder, located in an adjacent room. A television connected to the DVD recorder was used to monitor the behavior of the mice.

### Procedure

#### Experiment 1—Object recognition

Experimentally naïve female WT (*N* = 12) and GluA1^−/−^ (*N* = 12) mice were habituated to the empty test arena for 10 min per day for 3 d prior to the object recognition test. This procedure was used to reduce the possibility of context exploration interfering with object exploration (Besheer and Bevins 2000). Once a mouse was placed in the test arena the experimenter could observe the behavior of the mouse by watching the computer monitor which was located in a nearby room. The object recognition test commenced 24 h after the last habituation trial. For the exposure phase, two identical copies of the sample object (A1 and A2) were placed in the arena 10 cm away from the two adjacent corners of the north wall of the box and mice were allowed to explore the objects for 10 min. After an intertrial interval of 2 min, mice were returned to the test arena for 5 min for the test phase. During the test phase the arena contained a third, identical copy of the object used in the exposure phase (A3), and one novel object (B1). Each object was placed in a location previously occupied by the sample objects in the exposure phase. The orientation of the familiar object (A3) in the test phase was the same as in the exposure phase.

On each trial mice were placed into the arena facing the south wall and thus, facing away from the objects in the exposure and test phase. The arena and objects were wiped down with 70% ethanol between trials to minimize olfactory cues. Half of the mice were tested using the glass bottle and the tin can, and the remaining mice were tested using the plastic bottle and the wooden block. The object pairings were counterbalanced with respect to genotype. Also within genotype an equal number of mice were allocated one of the four objects as the novel object and either the left or right corner of the north wall as the location of the novel object in the test phase, in a factorial design.

Object exploration was measured as time spent with the nose <1 cm away from an object. Video footage of the exposure and test phase was scored by an observer who was blind with respect to the genotype of the animal and the object allocation during the test trial (i.e., novel or familiar).

No criteria were placed on exposure phase object exploration. However, if mice showed <1 sec total object exploration during the test phase, then they were removed from the analyses of the novel object preference in the test trial. All test trials were scored for the whole 5-min duration.

#### Experiment 2—Object recency

Experimentally naïve WT (female, *N* = 6; male, *N* = 6) and GluA1^−/−^ mice (female, *N* = 6; male, *N* = 6) were tested on the object-recency test. Mice received 3 d of habituation to the test arena, in the absence of any objects, in an identical manner to Experiment 1. Twenty-four hours after the last habituation trial mice received a 10-min exposure to two copies of the first sample object (A1 and A2). After a 2-min interval the mice were returned to the test arena and received a 10-min exposure to two copies of a second sample object (B1 and B2). The 5-min test trial commenced 2 min after the second exposure trial. In the test trial mice were exposed to new copies of both the first and second sample objects (A3 and B3). The allocation of the first and second sample objects and the location of the first and second sample objects in the test, were fully counterbalanced within each genotype.

#### Experiment 3—Yoked object recognition

Experimentally naïve WT (female, *N* = 6; male, *N* = 6) and GluA1^−/−^ mice (female, *N* = 6; male, *N* = 6) were tested on the object-recognition task. All procedures were identical to Experiment 1 except for the following details. The GluA1^−/−^ mice object exploration times in exposure and test phases were yoked to those of the WT mice. This was achieved by pairing WT mice and GluA1^−/−^ mice that were run under the same counterbalanced conditions (i.e., novel object allocation and novel object location). For a pair of mice, the WT mouse was run first and the experimenter recorded the total amount of time that the mouse spent exploring the objects during the exposure (i.e., A1 + A2) and test phases (i.e., A3 + B1). This was done by observing the behavior of the WT mice on the computer monitor that was located in a nearby room. The GluA1^−/−^ mouse was then subsequently run. The experimenter stopped both the exposure and test phases once the cumulative total object exploration times matched those of the WT mouse. If the GluA1^−/−^ mouse did not show a faster rate of object exploration than the WT mouse then the exposure phase was terminated after 10 min and the test phase after 5 min, thus matching the total time in the test arena for the WT mouse.

#### Experiment 4—Yoked object recency

Experimentally naïve WT (female, *N* = 12; male, *N* = 12) and GluA1^−/−^ mice (female, *N* = 12; male, *N* = 12) were tested on the object recency task. All procedures were the same as Experiment 2 except that the total object contact time for GluA1^−/−^ mice was yoked to that of WT mice for the exposure phases and test phase. For details of the yoking procedure, see Experiment 3, Methods.

#### Experiment 5—Context-dependent object recognition

Experimentally naïve WT (female, *N* = 4; male, *N* = 4) and GluA1^−/−^ mice (female, *N* = 4; male, *N* = 4) were tested for context-dependent recognition memory. Due to the task requiring mice to learn the context–object associations, mice received no preexposure to the contexts as this may result in latent inhibition of learning the contextual association (e.g., Killcross et al. 1998). Mice were exposed to two copies of object A (A1, A2) in context X and to two copies of object B (B1, B2) in context Y. Mice were exposed to each context for 10 min once per day for 4 d. The order of exposure to the two contexts alternated day by day (i.e., X-Y, Y-X, X-Y, Y-X), and within each day the two exposures were separated by approximately 2 h. On the fifth day mice received a 5-min test trial in each context. In the test trials mice were exposed to new copies of both objects A and B (i.e., A3 and B3, A4 and B4). Each test trial was separated by approximately 1 h. The identity of objects A and B (i.e., plastic bottle or wooden block), the location of the object that had not previously been paired with the test context (i.e., unpaired object; object A in context Y, and object B in context X), and the order of test trials in contexts X and Y were fully counterbalanced within each genotype. All other procedural details were the same as Experiment 1.

#### Experiment 6—Yoked context-dependent object recognition

Experimentally naïve WT (female, *N* = 8; male, *N* = 8) and GluA1^−/−^ mice (female, *N* = 8; male, *N* = 8) were trained on context-dependent object recognition. Procedural details were identical to those of Experiment 5 except that the amount of object exploration per exposure trial for GluA1^−/−^ mice was yoked to that of WT mice (for details of yoking procedure see Experiment 3). In the unyoked version of the context-dependent object recognition task (Experiment 5) it was found that, although GluA1^−/−^ mice showed greater object exploration than WT mice at the end of the exposure training trials, they actually showed less object exploration than WT mice at the start of the exposure training trials. Therefore, so that object exploration could be matched between genotypes for all training trials no cutoff time was applied during exposure training trials for GluA1^−/−^ mice. Similarly, there was no cutoff time for test trials, but all test trials were run for at least 5 min. Within sex and genotype the identity of objects A and B (i.e., plastic bottle or wooden block), the location of the object that had not previously been paired with the test context (i.e., unpaired object; object A in context Y, and object B in context X) and the order of test trials in contexts X and Y were fully counterbalanced.

### Analyses

Total time spent exploring the objects in the exposure and test phases and discrimination ratios were analyzed using either *t*-tests for comparison between two groups or multifactorial analysis of variance comparison of multiple factors. Where appropriate sex and genotype were included as between-subject factors and exposure phase trials were included as a within-subject factor. One-tailed, one-sample *t*-tests were used to assess whether preference for an object was significantly above chance (i.e., mean discrimination ratio >0.5). Also, one-sample *t*-tests were used to assess whether GluA1^−/−^ mice explored objects at a faster rate than WT mice in the yoked experiments (i.e., Experiments 3, 4, and 6), by comparing GluA1^−/−^ mice trial termination times with the set time limit for WT mice. A Type-1 error rate of 0.05 was adopted for all reported statistical comparisons.

## Footnotes

4 Retrospectively yoking the exploration of GluA1^−/−^ mice to that of WT mice in tests trials for Experiments 1 and 2, by analysis of the videotaped trials, failed to change the pattern of results. Thus, the discriminations ratios for GluA1^−/−^ mice based on the yoked levels of exploration in the test phase did not significantly differ from WT mice (see Supplementary Results and Supplementary Figure 1). It remains possible, however, that increased levels of exploration in the exposure phase, rather than in the test phase, may have masked a potential deficit in GluA1^−/−^ mice in Experiments 1 and 2.
